# A perspective on developing solid-phase extraction technologies for industrial-scale critical materials recovery

**DOI:** 10.1039/d2gc00347c

**Published:** 2022-03-11

**Authors:** Aaron Brewer, Justyna Florek, Freddy Kleitz

**Affiliations:** Department of Inorganic Chemistry – Functional Materials, Faculty of Chemistry, University of Vienna Währinger Strasse 42 1090 Vienna Austria aaronbrewer5@gmail.com freddy.kleitz@univie.ac.at

## Abstract

Critical materials (CMs) are a group of elements that have been determined to be important for the modern economy, but which may face current or potential supply limitations. Some examples of metals that have received the CM designation include the rare earth elements, indium, gallium, and lithium. The last decade has seen a major push for the development of new and improved technologies for the recovery and purification of CMs from various traditional and non-traditional resources in an effort to diversify supply. Solid-phase extraction (SPE) is one broad category of these experimental extraction technologies. SPE involves the application of a solid material to preferentially retain in the solid phase one or more specific components of an aqueous solution, leaving the other components behind in the aqueous phase. A wide range of different sorbents has been used for SPE, and many offer significant potential advantages, including low cost, low environmental impact, and high customizability. Hierarchically porous silica monoliths are one example of a cutting-edge sorbent that provides a durable, high surface area foundation that can be functionalized with a variety of targeted ligands for the selective extraction of specific CMs. Despite impressive recent advances in SPE, there remain areas for improvement that are common across the discipline. To demonstrate the practical viability of these innovative CM recovery systems, future SPE studies would benefit from devoting additional focus to the scalability of their material, as well as from focusing on real-world feedstocks and conducting techno-economic analyses and environmental impact studies.

## Abstract 2

Solid-phase extraction offers numerous advantages for critical materials recovery, and research in the field would benefit from increased focus on environmental impact, techno-economic assessments, and process scalability with real-world resources.

## Graphical abstract

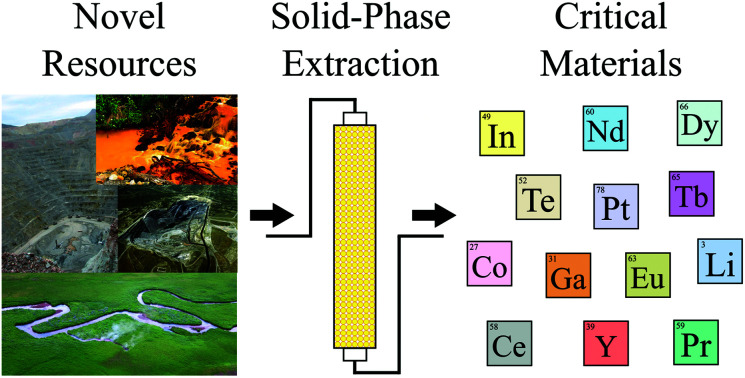

## 1. Introduction

Critical materials (CMs) are elements considered to be highly important to the modern international economy but which face widespread supply risks, often due to political factors. The specific elements considered to be CMs may change over time; however, the U.S. Department of Energy (DOE) and the European Commission both currently include a range of rare earth elements (REEs; *e.g.*, dysprosium, neodymium, and terbium) as well as several other metals, like lithium (Li), indium (In), and cobalt (Co).^1,2^ For a full list of the identified critical materials, see the U.S. DOE Critical Materials Strategy Report^1^ and the European Commission's Study on the EU's list of Critical Raw Materials.^2^ In application, many CMs are especially important for the development and expanded use of green energy technologies. Tellurium (Te), for example, is a central component of advanced photovoltaic cells, while large amounts of neodymium (Nd) are used in both electric automobile motors and wind turbines. Unfortunately, in some cases, the positive environmental implications that can come from the use of a given CM are, at least in part, countered by the harmful impacts of the extraction and purification process for that metal. Lithium supply and disposal, for instance, has become notorious for its damaging effects on the environment.^3^ There has been a recent surge in research dedicated to mitigating the supply risks and environmental hazards associated with different CMs, particularly in the EU, North America, and Japan. Common avenues of investigation include designing new and improved techniques for recovering CMs from established resources, assessing the viability of previously unexploited or underexploited primary CM feedstocks, developing methods to recycle CMs from waste products, and replacing CMs with functionally similar metals that do not face the same supply constraints. Innovation in the methods available for the extraction and purification of specific CMs will make the acquisition of these metals simpler, more economical, and more environmentally friendly.

Critical materials supply chains are often dominated by just a few different sources and processing techniques that are well-established within the industry, even though numerous other potential resources and methods may be available. Indeed, metals commonly receive the “CM” designation because the established resources that supply them are somehow inadequate for the widespread and large-scale production necessitated by increasing international demand. Among the proven CM resources, primary feedstocks may be exploited specifically for CM recovery, like for example, the REE ores at the Bayan Obo mine in China^4^ or the Li-rich pegmatites at the Greenbushes mine in Australia^5^ (Table 1). However, in other cases, CMs are recovered as a byproduct of the supply chain of some other material, such as In extraction from sulfides being exploited for copper and zinc, or gallium (Ga) extraction from bauxite being exploited for aluminum (Table 1). In addition to these existing CM resources, there are various new feedstocks which might provide a means to diversify CM supply and decrease our dependence on sources with a high environmental cost. Recycled feedstocks can be particularly attractive due to their high CM contents in desirable concentration ratios, in addition to the positive environmental implications of developing a closed-loop supply chain. Electronic waste, both from consumers and from industrial applications, is especially promising; for instance, NdFeB magnets, which are important components of electronic devices like hard discs and speakers, contain high concentrations of Nd as well as smaller amounts of other REEs.^6^ Liquid crystal displays (LCDs), on the other hand, are a candidate for indium extraction,^7^ while tellurium could potentially be recycled from the photovoltaic cells that drive their demand.^8^ In addition to recycled electronic materials, the myriad other underutilized potential feedstocks include spent catalysts,^9^ acid mine drainage,^10^ geothermal fluids,^11^ mining wastes like red mud^12,13^ and coal byproducts,^14,15^ and many others (Table 1). This wide array of abundant and easily obtainable resources could help to mitigate the existing CM supply limitations and environmental impacts if the appropriate extraction and purification systems can be developed.

**Table 1:** A selection of critical materials with their primary and some potential feedstocks

Critical materials	Primary feedstocks	Potential feedstocks
REEs	Bastnäsite	Acid mine drainage
	REE-phosphates	Coal byproducts
	Ion-adsorption clays	Red mud
		Electronic waste

Indium	Sulfides mined for Cu and Zn (*e.g.*, sphalerite)	Manufacturing waste
		Electronic waste

Gallium	Red mud (bauxite mined for Al)	Manufacturing waste
	Sulfides mined for Zn	Electronic waste
		Coal byproducts

Lithium	Pegmatite (spodumene, lepidolite, petalite)	Seawater
	Natural brines	Li-battery waste
		Select clay minerals

Cobalt	Sedimentary Cu and Ni deposits (*e.g.*, laterite)	Manganese nodules
	Magmatic Ni–Cu–Co deposits (*e.g.*, pentlandite)	Select iron ores

Tellurium	Porphyry deposits mined for Cu	Recycled solar cells
		Select gold ores

Critical materials recovery is typically a complex, multi-stage process that varies significantly between different CMs and different feedstocks. The concentration of a CM in a given material can range from more than 50 wt% all the way down to sub-ppb levels. Concentrating and purifying a CM from among the abundant contaminating components in an efficient, green way is therefore a significant challenge. For solid materials, especially primary mineral resources, there is often an initial physical beneficiation process (*e.g.*, flotation or magnetic separation) to remove gangue minerals and pre-concentrate the CM. Thorough chemical separation is typically achieved through a series of one or more pyrometallurgical, hydrometallurgical, and/or electrometallurgical processes. In many cases, these different techniques offer complementary advantages and are therefore employed together as components of the overall multi-step extraction and purification scheme. Pyrometallurgy involves the application of a thermal process, such as roasting or smelting, to purify a given component of a material. Nickel, for instance, is frequently produced through the reductive smelting of laterite rocks.^16^ Hydrometallurgy, on the other hand, involves the separation of metals in aqueous solution at relatively low temperatures. Leaching, precipitation, solvent extraction, and supported liquid extraction are commonly used methods in this category. The purification of REEs, for instance, is today primarily accomplished through liquid–liquid extraction (LLE), which exploits small differences in the chemical behavior of the REEs to partition them between immiscible aqueous and organic solvents over many sequential steps.^17^ Electrometallurgy, including electro-winning and electro-refining, employs an electric potential difference to facilitate reactions in aqueous solutions, as in hydrometallurgy, or in molten salts, as in pyrometallurgy. It therefore most often serves as a complementary technique to these other methods.^16^ Significant research and development is being devoted to designing and implementing new and improved extractive metallurgy techniques for CMs both in industry and in academia. Regardless of which aspect of the process is the focus of a given investigation, it is extremely important to consider how that one component or technique fits into the complex system as a whole.

The present Perspective will focus specifically on solid-phase extraction (SPE), a type of hydrometallurgical purification technique that is at the center of significant CM research today, especially in the context of non-traditional CM resources and environmentally-friendly recovery systems. The Perspective will briefly discuss the state of the field, including the present advantages of SPE as well as some challenges facing its widespread adoption at the industrial scale. Hierarchically porous silica monoliths will be presented as a representative case study. We will then identify some ways in which academic research efforts into SPE could better address the promising real-world applications of the technique moving into the future.

## 2. Solid-phase extraction

**Fig. 1:**
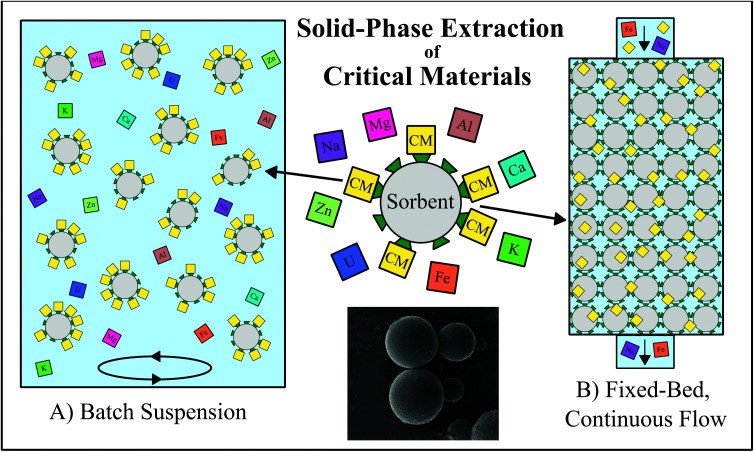
Schematic representing the application of solid-phase extraction (SPE) techniques for the selective recovery of critical materials (CM; yellow squares). (A) Batch reaction system in which the sorbent (grey circles with green triangles representing surface functional groups) is suspended and agitated within a liquid feedstock to preferentially retain CMs in the solid phase, leaving the contaminating metals (multicolored squares) behind in the liquid phase. (B) Fixed-bed, continuous flow system in which the liquid feedstock in passed through an immobile mass of the extractant, again preferentially retaining CM in the solid phase while the contaminating metals exit the column with the liquid feedstock. The SEM image at bottom center is an example microbe bead sorbent, reproduced from ref. 20 with permission from ACS Publications, 2019.

Solid-phase extraction (SPE) is one promising hydrometallurgical method for which numerous experimental techniques have recently been proposed. SPE has been used as a metal separation method for decades, but typically only on relatively small scales to produce an especially high purity product or to accomplish exceptionally difficult separations. Even as early as the Manhattan Project, for example, SPE was employed to separate the lanthanides in reactor fission products in preparation for analysis.^18^ The technique operates on the principle of applying a reactive solid material to selectively retain desired metals from an aqueous solution, such as a mineral or waste leachate. The chosen solid will typically have a high affinity for the adsorption of a specific metal or group of metals compared to any co-existing species. During the recovery process, this solid extractant, or sorbent, can be either dispersed into a volume of the feedstock in a batch reaction or packed into a fixed-bed column through which the feedstock will flow (Fig. 1). Both batch and column systems are most often designed so that, at a certain controlled set of conditions, the contaminating feedstock components will remain in the liquid phase, while the desired components are adsorbed and concentrated in the solid phase, although in some cases, the solid sorbent can be designed to extract specific contaminating metals. The liquid phase is then separated from the sorbent, and the sorbed metals are recovered from the solid through desorption with a chemical eluent. Both the adsorption and desorption steps may contribute to the concentration and purification of the desired element(s) from a large volume of the bulk feedstock into a smaller volume of eluent. Multiple SPE procedures may be run in sequence, and/or in conjunction with other types of extraction methods, to achieve high purity mono- or multi-element products. With proper sorbent selection, and in conjunction with proper process design, an SPE system is capable of increasing the concentration and purity of a given metal by orders of magnitude, even from challenging, non-traditional feedstocks.

There is tremendous variability amongst SPE systems, and each different extractant has advantages and disadvantages for metal recovery and purification purposes. Modern experimental sorbents range from prawn carapace^19^ to bacteria,^20,21^ clay minerals,^22,23^ mesoporous silica,^12,24^ carbon nanotubes,^25,26^ and many others. The ideal adsorbent would exhibit both a high capacity and a high selectivity for the desired metal or metals and would be inexpensive, readily available/scalable, mechanically and chemically stable, and environmentally-friendly. Table 2 includes a varied selection of some of the SPE materials that have been used for CM extraction, and numerous existing publications provide more comprehensive overviews and comparisons of the properties of these experimental materials.^16,27–31^ The aim of this Perspective is rather to discuss the characteristics and direction of the field as a whole, while proposing some ways in which our fundamental approach to developing SPE systems could be improved in the context of real-world applications.

**Table 2:** A selection of experimental SPE sorbents for CMs from among the hundreds that have been tested, including their targeted CMs and feedstocks. The feedstocks labeled ‘Artificial’ are simple laboratory solutions, while the named feedstocks are real-world solutions or close synthetic approximations

Experimental extractants	CM	Feedstocks
*E. coli*	REE	Fly ash,^61^ geothermal fluids,^11^ electronic waste^20^
Crab shells	Co	Artificial^62^
	REE	Artificial^63^
Sawdust	In	Industrial wastewater^64^
	REE	Artificial^19^
Algae	Co	Artificial^65^
	Ga	Industrial wastewater^66^
	In	Artificial^67^
	REE	Acid mine drainage,^68^ seawater^68^
Chitosan	Co	Artificial^39^
	Ga	Artificial^43^
	Li	Artificial^37^
	REE	Waste phosphors^69^
Mesoporous silica	Co	Artificial^70^
	REE	Artificial^12^
Hierarchically porous silica monoliths	REE	Bauxite^42^
Mesoporous carbon	Co	Artificial^71^
	Ga	Artificial^72^
	In	Artificial^73^
	REE	Artificial^44^
Carbon nanotubes	Ga	Fly ash^15^
	In	Artificial^74^
	Li	Artificial^26^
	REE	Artificial^75^
	Te	Artificial^25^
Graphene oxide	Co	Artificial^76^
	Ga	Artificial^77^
	Li	Lake Brine^38^
	REE	Artificial^78^
Zeolite	Co	Artificial^79^
	Ga	Bauxite^13^
	Li	Geothermal fluids^80^
	REE	Artificial^81^
Clays	Co	Artificial^22^
	REE	Artificial^23^
Zero-valent iron	Co	Industrial wastewater^82^
	In	Industrial wastewater^82^
	Ga	Industrial wastewater^82^
	REE	Acid mine drainage^83^
	Te	Artificial^84^
Manganese oxide	Co	Artificial^85^
	Li	Seawater^86^
Titanium oxide	Li	Shale gas wastewater^87^
	REE	Artificial^88^
	Te	Industrial wastewater^89^

### 2.1 Advantages

In principle, solid-phase extraction presents numerous potential advantages as a metal recovery and purification technique. SPE can be a low-cost system that is highly adaptable and well-suited for a variety of feedstocks, especially non-traditional feedstocks, while also limiting the severe environmental impact that is unfortunately common for extractive metallurgy processes.

Solid-phase extraction systems can be quite cost effective, in some cases because the sorbents themselves can be cheap to produce or acquire, but also because the solid materials tend to be highly reusable.^31^ Although some sorbents are highly engineered substances synthesized in a laboratory at relatively high expense, others already exist in large quantities and need only be diverted for SPE use. Waste products from the agriculture (*e.g.*, orange peels^19^) and fisheries (*e.g.*, fish scales^19^) industries are prime examples of inexpensive, readily available sorbents. These types of wastes are continuously generated in large volumes, and there is an existing demand for their disposal. Industrial SPE operations could help to fill this demand, acquiring the sorbent that they need at minimal expense, while also providing a means for the waste materials to be used productively. Many solid-phase extractants, as an additional advantage, can retain their function over multiple adsorption/desorption cycles.^31^ The repeated process of CM retention as the sorbent is exposed to the feedstock followed by the elution and recovery of the CM, in many cases, has only a minor effect on the surface chemistry and physical structure of the extractant.^31^ Even extractants with a relatively low recovery capacity could therefore potentially process a large amount of a given CM over time without needing to be replaced. This combination of inexpensive materials and reusability makes SPE a long-term, more sustainable solution for CM recovery while also limiting materials costs.

**Fig. 2:**
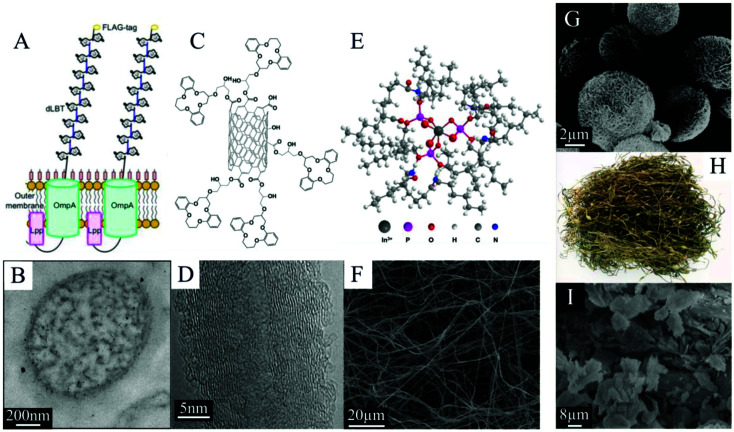
(A) Schematic representing the surface display of lanthanide binding tags on engineered *E. coli*, reproduced from ref. 20 with permission from ACS Publications, 2019; with (B) a TEM image of one cell used for REE extraction, reproduced from ref. 33 with permission from ACS Publications, 2017. (C) Schematic representing crown ethers immobilized on carbon nanotubes, with (D) a SEM image of carbon nanotubes used for Li extraction, both reproduced from ref. 26 with permission from Elsevier, 2015. (E) Schematic of nylon nanofibers modified with DEHPA, with (F) a SEM image of the spun nylon used for In extraction, both reproduced from ref. 90 with permission from Elsevier, 2019. These three surface-modified materials can be compared with sorbents with little or no modification that do not employ designer ligands for CM recovery and purification, such as (G) SEM image of oxidized microsphere flower carbon reproduced from ref. 91 with permission from ACS Publications, 2021; (H) photograph of brown algae, reproduced from ref. 67 with permission from Springer Nature, 2019; and (I) SEM image of zeolite, reproduced from ref. 81 with permission from MDPI, 2021. Each of these sorbents has been proposed for CM recovery.

**Fig. 3:**
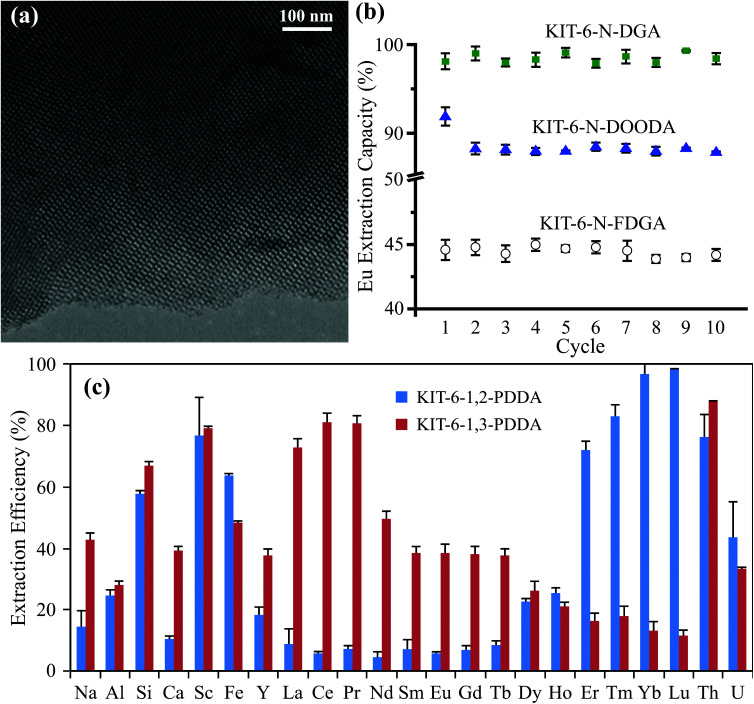
(a) TEM image of a representative mesoporous silica (KIT-6 functionalized with DGA) showing the ordered pore structure, reproduced from ref. 24 with permission from ACS Publications, 2020. (b) Extraction efficiency of europium (Eu) over ten adsorption/desorption cycles by mesoporous silica functionalized with various ligands, reproduced from ref. 47 with permission from RSC Publishing, 2015. (c) Batch extraction efficiency for REEs and competing metals from a real bauxite residue using mesoporous silica functionalized with phenylenedioxy diamide (PDDA, with two different bite angles), reproduced from ref. 12 with permission from ACS Publications, 2019.

SPE also has the potential to provide excellent recovery selectivity for many different metals from many different feedstocks due to the wide range of functional groups that the material surfaces can exhibit both naturally and through artificial surface modification (Fig. 2).^31^ In many cases, with proper process design, an SPE system can exploit the sorptive characteristics of even simple, abundantly occurring functional groups, like silanols^32^ and carboxyls,^21^ to achieve good separation performance. However, it may often be desirable to improve the surface functionality of a sorbent in order to increase adsorption capacity and selectivity, and these targeted adjustments are readily achievable with SPE materials. Microbes, for example, can be bioengineered to express particular ligands, like lanthanide binding tags,^33^ on the cell surfaces (Fig. 2). Silicas, carbons, and other synthetic materials (*e.g.*, polymers and various metals) are frequently modified with designer ligands through grafting or by employing a one pot synthesis procedure.^34,35^ Numerous different ligands have been proposed for CM extraction, including lanmodulin^36^ and phenylenedioxy diamide^12^ for the REEs (Fig. 3), crown ethers for Li,^26,37,38^ and maleic acid for Co.^39^ The sorbent surfaces are therefore capable of being precisely optimized for specific metal production goals and specific feedstocks, and this customizability makes SPE an extremely flexible and adaptable method for CM recovery and purification.

Indeed, the ability to function with a wide range of atypical feedstocks, especially low-grade feedstocks, is a major advantage of SPE systems. The existing technologies in use today for CM separation and recovery, with the exception of methods that generate CMs as a byproduct, typically require relatively high-grade feedstocks to operate efficiently. The bastnaesite and monazite ores, for example, from which the majority of REEs are currently produced, contain 70–74 and 35–71 wt% rare earth oxides, respectively,^40^ and feedstocks with ppm or ppb levels of REEs are not usually considered viable for industrial-scale extraction. However, with continuous or semi-continuous flow SPE systems, many liters of feedstock can be rapidly passed through small volumes of a fixed-bed solid sorbent. The sorbent would preferentially retain the CMs, and with the appropriate system design, the CMs in successive volumes of feedstock could competitively displace adsorbed competing metals from the previous volumes. Note that these column operations are largely passive processes; a liquid feedstock is simply exposed to the extractant, and then periodically the CMs are collected and the sorbent is regenerated through an elution and conditioning step. SPE therefore has the unique potential to enable added-value CM recovery at sites not dedicated to CM production since, in principle, minimal additional equipment, chemicals, and facilities are necessary once the resource is in liquid form. Acid mine drainage and geothermal fluids, for example, can contain low, but non-negligible, CM concentrations and are already subject to human intervention at a large-scale. A SPE step could potentially be incorporated into the existing processes to provide small-scale CM production, which would occur in addition to the existing aims of the operation. The CM yields would be small, but costs could also be quite low given that much of the required infrastructure would already be in place. These sorts of added-value systems are still largely speculative; however, the ability for a stable, solid sorbent to passively extract CMs from a large volume of low-grade solution with high selectivity could enable a breadth of promising and unique applications for SPE.

For many of the same reasons that SPE can be a highly cost effective and efficient technique, it also has the potential to recover CMs with a low environmental impact. The waste materials that can be repurposed as SPE sorbents present an obvious environmental advantage, in that they both do not require material and energy inputs to produce and that they impart value to an unwanted waste that previously just required treatment and disposal. Additionally, many sorbents that are produced intentionally for SPE, like microbes, do not require hazardous materials for production and do not typically involve significant volumes of hazardous chemicals in the extraction process. Other hydrometallurgy techniques, like LLE, do require organic solvents, so this attribute of SPE systems may represent a major advantage over existing systems, though unfortunately, little quantitative data is available so far which directly compares the environmental impact of the different systems. The reusability of SPE sorbents further decreases the necessary material and energy requirements for a given CM yield. Additionally, the customizable surface chemistry and added-value extraction capabilities of SPE sorbents enables their use with many potential new feedstocks. Indeed, the development and widespread adoption of SPE technologies could make a varied selection of novel feedstocks viable for CM extraction, especially low-grade feedstocks which have never before been feasible options. This advance would diversify the CM feedstock options available for exploitation and could limit our dependence on some resources that are known to have a high environmental impact. In particular, SPE could help enable CM recycling from various waste products, from electronics waste to fly ash, moving the industry towards a more sustainable, potentially even closed-loop, CM supply chain.

### 2.2 Current limitations

Despite the many potential advantages of SPE, there are some possible disadvantages and limitations of the technique. Some of these disadvantages are simply a function of the state of the field at the present time, and these concerns will be addressed in the section 4. However, some limitations may be characteristic of many SPE systems in general.

The choice of available SPE sorbents is highly variable and highly customizable; however, the more advanced materials tend to come with increased cost. Salmon milt,^41^ for instance, could be used as a green CM extractant in some instances. But, if the situation calls for a sorbent that does not restrict fluid flow when packed into a column system even at high flow rates, a more specialized, highly engineered material (*e.g.*, a hierarchically porous silica monolith;^42^ see section 3) would be required and would likely be substantially more expensive, especially at a large scale. Similarly, the salmon milt is adsorptive,^41^ but may not be highly selective for a particular metal of interest. To achieve exceptional adsorption selectivity, the sorbent would likely need to be functionalized with a targeted ligand (*e.g.*, an ion-imprinted ligand^15,38,43^), and these highly-engineered materials would likely be much more expensive to produce at that large scale. Lastly, certain feedstock characteristics, like low pH and high temperature, may limit the effectiveness of many sorbents, and may even result in their degradation over time. Advanced materials (*e.g.*, mesoporous carbon, which can continue to perform well at pH < 3 ^44^) have been developed that can function in these harsh conditions, but again the more highly engineered materials typically involve increased costs and environmental impact. While it may be possible to develop a sorbent that exhibits the most important advantages of advanced synthetic functional materials while also being green and inexpensive to produce, at present there is often a trade-off between cost, environmental impact, and high-level extraction performance.

The adsorption capacity of many solid-phase extractants may face some practical challenges with high concentration feedstocks. Published capacities typically range from <1 to 300 mg CM per g sorbent,^16,27–31^ given that a fraction of the sorbent mass does not contribute to CM adsorption, even for the highest surface area materials. To extract at one time 1 kg of a CM could therefore require hundreds of kg of sorbent, which is unlikely to be feasible in every situation. Of course, the excellent reusability of these sorbents means that a mass of a given extractant could extract much more than its sorption capacity of a CM over multiple adsorption/desorption cycles during its effective lifetime. Recovering 1 kg of a CM from a low-grade feedstock (low- or sub-ppm) could also mean that more than hundreds of thousands of kg of that feedstock were passively processed. However, high-grade feedstocks often contain a given CM on the order of wt% (*e.g.*, the traditional REE ores^40^). Depending on sorption capacity, a truly excessive amount of extractant could theoretically be required to process even small batches of these high-grade resources. In such cases, LLE, or another technique, may be a more efficient means of CM recovery. Maximizing adsorption capacity will broaden the range of feedstocks for which SPE could be viable, though very high-grade feedstocks may always present a challenge for the technique.

**Fig. 4:**
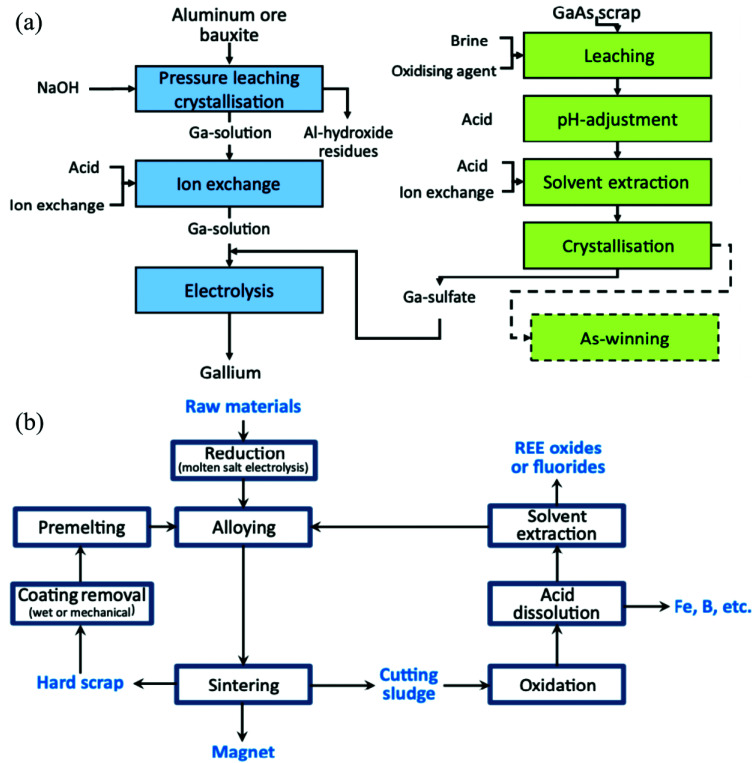
(a) Process scheme for the recovery of Ga from common feedstocks. (b) Process scheme for the recycling of REEs from permanent magnet waste. Both panels reproduced from ref. 92 with permission from Elsevier, 2014.

Finally, it is important to recognize that, given its advantages and its limitations, SPE is not a panacea that eliminates the need for other recovery and purification techniques. The most cutting-edge feedstock-to-product process designs for SPE still often include a roasting (pyrometallurgy) step, for example, to prepare the solid for leaching and/or to convert the CM into its final, marketable form.^16^ These other extractive metallurgy techniques can be valuable components of an SPE system, and SPE can also be a valuable component of other systems (Fig. 4). For instance, SPE could perhaps preconcentrate CMs from a low-grade feedstock, or selectively remove a particularly troublesome contaminant, before final recovery and purification is accomplished *via* LLE. R&D efforts are always looking to supplant the existing industry standards for a given process with something more efficient, cost effective, and/or environmentally friendly, and indeed, SPE may offer a favorable alternative to other extractive metallurgy techniques in some cases. However, these different techniques may just as often work in conjunction with each other, and advances in one area could provide a benefit to the others as well.

## 3. Solid-phase extraction using hierarchically porous silica monoliths: a case study

Hierarchically porous monoliths are one promising sorbent currently in development for practical CM recovery, largely on the basis of their tunability, flexibility with various feedstocks, and compatibility with continuous and semi-continuous flow extraction systems. This family of materials provides an interesting case study that is representative, in many ways, of the field of solid-phase extraction for CMs at large.

Hierarchically porous silica monoliths are materials similar in some ways to the more well-known ordered mesoporous silica powders (*e.g.*, MCM-41, SBA-15, and KIT-6), and they exhibit many of the same advantageous properties. The monoliths and powders are formed based on a cooperative (self)-assembly between an inorganic precursor (*e.g.*, tetraethylorthosilicate or tetramethylorthosilicate) and one or more organic templates (*e.g.*, triblock copolymers and cationic surfactants).^12,24,32,34,45–47^ After the template is removed, the powders that remain exhibit an ordered array of well-calibrated mesopores (2–50 nm in diameter), providing exceptionally high surface area and high pore volume for adsorption (Fig. 3).^12,24,32,34,45–47^ These mesoporous sorbents have been shown to be highly reusable, able to endure multiple adsorption/desorption cycles with minimal loss of function (Fig. 3).^12,34,45,47,48^ The mesoporous silica, both in powder and in monolith form, can furthermore be functionalized with a variety of additional ligands, through simple organic reactions, co-condensation, or silanization, depending on the material, in order to increase adsorption and to adapt the materials for targeted CMs extraction objectives.^24,34,45–47^ Specific ligands have been developed, for example, for the rare earth elements (REEs), which can improve both the adsorption selectivity and the overall adsorption capacity of the powders.^24,34,45–47^ On the basis of these characteristics, mesoporous silica materials provide a highly promising basis for SPE applications.

**Fig. 5:**
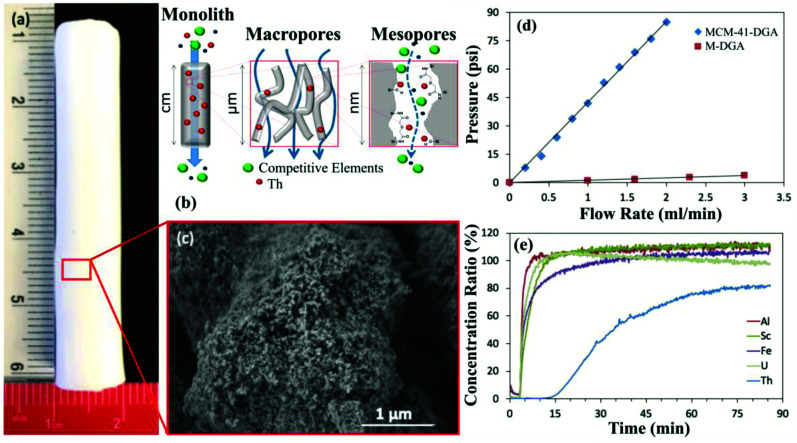
(a) Photograph of a hierarchically porous silica monolith. (b) Schematic illustrating the selective extraction of thorium (Th) from a mixed metal solution as it passes through the macro- and mesopores of a monolith sorbent. (c) HRSEM image of the porous structure of the silica monolith in panel (a). (d) Plot showing the pressure increase with flow rate for mesoporous silica powder (MCM-41-DGA) compared to a hierarchically porous silica monolith (M-DGA). (e) Breakthrough column results for the selective extraction of Th, a common radioactive contaminant in REE feedstocks. Note the rapid breakthrough of most tested metals, while Th breakthrough is delayed due to preferential retention in the silica monolith column. All panels reproduced from ref. 42 with permission from RSC Publishing, 2019.

Hierarchically porous monoliths broadly offer many of the same promising sorption characteristics as mesoporous powders, with the additional advantage of facilitating rapid mass transfer of the feedstock owing to the presence of large macropores. The monoliths are synthesized based on the same principles as the mesoporous powders; however, the porous silica structure is produced as a single highly structured mass rather than as a collection of individual particles (Fig. 5).^42,49–51^ The monoliths typically exhibit a hierarchically porous structure with both meso- (2–50 nm in diameter) and macropores (>50 nm in diameter) and can be produced in a variety of shapes and sizes (Fig. 5).^42,49–51^ The pore characteristics, including size, shape, connectivity, and wall thickness can all be controlled by fine-tuning the synthesis parameters.^42,49–51^ The resulting highly interconnected network of macropores permits feedstock fluid flow through the monolith sorbent with nominal resistance.^42,52^ While a volume of mesoporous powder will restrict fluid flow when packed into an extraction column, causing elevated column pressure as flow rate increases, an equivalent volume of sorbent in the form of a hierarchically porous monolith will cause only minimal increases in column pressure (Fig. 5).^42,52^ In principle, a large volume of feedstock could be very rapidly passed through these high surface area monoliths, maximizing feedstock exposure to the adsorptive surfaces, which in turn maximizes CMs recovery potential. The monoliths are therefore expected to be more compatible with industrial-scale SPE recovery systems, which are commonly based around this type of continuous or semi-continuous flow design. The excellent mass transfer, combined with the many existing advantages of mesoporous silica (*i.e.*, high surface area, durability, flexibility, *etc*.), makes hierarchically porous monoliths extremely promising for CM extraction.

In an effort to exploit these advantages, hierarchically porous silica monoliths are beginning to be assessed for practical CM extraction and purification applications. To provide an initial proof of principle, unfunctionalized silica monoliths were applied to selectively remove thorium (Th) from bauxite residue leachates.^42^ Thorium is well-known as a problematic contaminant in REE extraction efforts, and its elimination, leaving the REEs behind in the aqueous phase, is a major step towards REE purification for certain feedstocks (Fig. 5). The monolith columns were able to extract >80% of the Th in the leachate, and were observed to retain their function over at least 10 adsorption/desorption cycles.^42^ Furthermore, the monoliths exhibited minimal increase in column pressure during operation, in contrast to a comparable mesoporous silica powder, confirming the exceptional performance of the monoliths in continuous-flow systems.^42^ Hierarchically porous silica monoliths functionalized with targeted ligands were also used for the highly selective adsorption of palladium (Pd)^53,54^ and cobalt (Co)^55^ in model solutions, along with several other non-CMs, but desirable, metals, including gold^56^ and silver.^57^ The behavior of these different monoliths was again characterized by repeatable performance over multiple recovery cycles as well as excellent function under continuous flow.^53–55^ These results demonstrate the fundamental potential of hierarchically porous silica monoliths for CM extraction, though additional development is required before this technology will be ready for industrial use.

Perhaps the most significant challenge remaining for the real-world application of hierarchically porous silica monoliths is scaling the materials up to the required sorbent volumes. The monoliths tested for CM extraction have typically been up to ∼1 cm in diameter and several cm in length.^42,53–55^ This size is clearly insufficient for processing many liters of a feedstock to produce kilograms of a purified CM product. The synthesis of larger silica monoliths is not necessarily straightforward though, as the monoliths, especially the larger monoliths, are fragile and prone to cracking, particularly during drying. Numerous studies have offered potential solutions to this issue, ranging from coating the monoliths in paraffin oil during drying^58^ to supercritical drying techniques.^59^ Cladding the larger monoliths, and to a lesser extent the smaller monoliths, for column applications without causing breakage has also proven to be challenging. Proposed solutions commonly include heat-shrink tubing and resin coatings.^50^ However, to date there has been, to our knowledge, no industrial-scale silica monoliths synthesized and applied for CM recovery purposes. Despite all of their advantages, the consistent production and use of large-scale hierarchically porous silica monoliths must be demonstrated before they can be considered for real-world CM extraction operations.

Several other aspects of the technological development of hierarchically porous silica monoliths must be further investigated before the real applicability of the sorbent can be assessed. First, the extraction performance of the monoliths must be tested with additional real-world feedstocks. CM sorption by these monoliths has, so far, primarily been measured using controlled, artificial solutions, which provide only a limited picture of the challenges facing extraction from real-world feedstocks, especially since the sorbents themselves will likely be modified, for example through targeted ligand grafting, to maximize performance for a particular feedstock. Second, the cost of synthesizing the monoliths and conducting extraction operations using them on a large scale has not been assessed. It is unknown under what circumstances this SPE technology could be economically viable, and promising feedstocks and potential areas of improvement in terms of cost reduction and process design have not been identified. Finally, no studies have been undertaken to determine the potential environmental impact of monolith production and use, which will be vital if this technology is going to contribute to the greener CM extraction systems of the future. Hierarchically porous silica monoliths show great potential as solid phase extractants for CMs; however, there are clear areas in which the development of the technology must be improved. These same shortcomings are common to many of the experimental SPE materials currently under development, as will be discussed in more detail in the following section.

## 4. Future directions

The development of SPE materials and methods for CM recovery is applied science, and ongoing academic research could do more to embrace the applied nature of the field. SPE research does, of course, present opportunities to further our understanding of fundamental chemical and physical processes that may have broad implications; however, the motivation for pursuing advances in SPE is often based on the real-world need to find easier, more economical, and more environmentally-friendly ways to secure supplies of a particular material. Despite claiming this practical motivation, reported findings in the field often fail to fully address their real-world applications; specifically, more focus should be devoted to the scalability, economic feasibility, and environmental implications of emerging SPE technologies. All three of those factors are critical for determining the practical utility of new developments in SPE.

Scalability is often as important as fundamental extraction performance when it comes to the real-world application of a SPE methodology. An extractant may have remarkable recovery capacity and selectivity for a desirable CM, but if it cannot feasibly be produced or acquired in quantities greater than a few grams at a time, then it is simply not yet a viable material regardless of its performance. On the other hand, an abundant material with great performance may also not be scalable if it is incompatible with the basic techniques that are practical for industrial-scale CM extraction operations. The most common examples of this issue are materials that limit or entirely obstruct fluid flow when packed into fixed-bed columns, making them incompatible with continuous and semi-continuous flow systems. The production of sorbents and the ways that they can be used in real-world recovery methodologies are fundamental considerations for SPE research. SPE studies should assess how these factors affect the scalability of their product alongside its basic extraction performance to ensure that it has actual practical implications in addition to promising chemical characteristics.

**Fig. 6:**
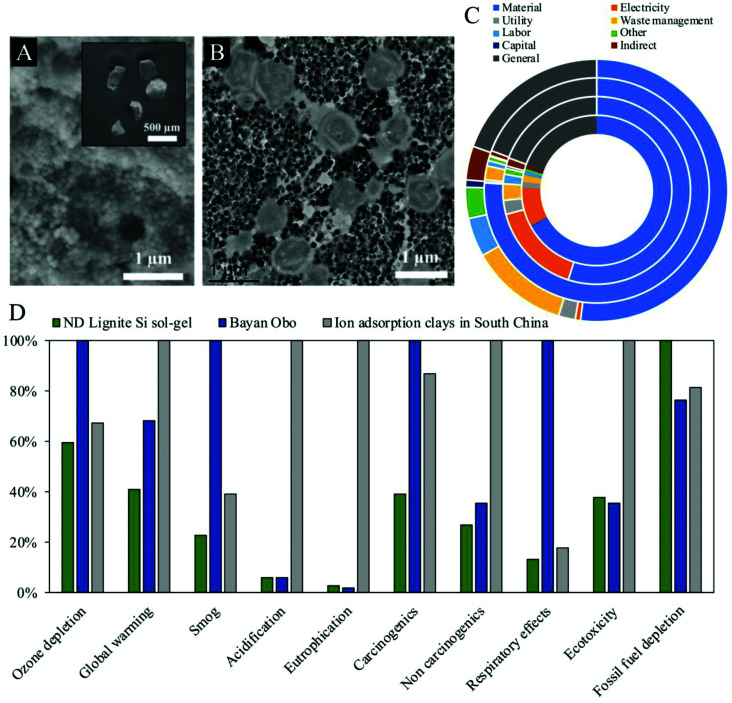
(A) SEM and (B) TEM images of encapsulated microbes for the extraction of Sc from lignite coal, reproduced from ref. 61 with permission from ACS Publications, 2021. The process involves leaching of the coal followed by two distinct column extraction steps. (C) REE extraction cost breakdown for two biosorbents with lignite (two outer rings) and fly ash feedstocks (two inner rings), reproduced from ref. 93 with permission from ACS Publications, 2020. (D) Comparison of environmental impacts for existing REE extraction techniques in China (Bayan Obo and ion adsorption clays) and an experimental biosorption approach (ND lignite), reproduced from ref. 93 with permission from ACS Publications, 2020.

Techno-economic analyses involving the assessment of real-world feedstocks are also critical components of CMs research that are often absent in SPE studies. Again, a material may have excellent extraction performance, but if it would cost more to produce and to operate than the expected yield from the recovered CM, then it is not yet a viable extractant. To accurately assess expected yields, it is vital to conduct experimental extractions with the specific real-world feedstock target(s), or at least close synthetic approximations. Real feedstocks exhibit highly variable compositions, including different pH, a wide range of competing metals, and diverse anion and ligand contents, all of which can strongly affect CM sorption behavior. Experiments with mono-element solutions or other highly simplified feedstocks may be informative regarding the basic properties of the sorbent, but they are not sufficiently realistic to be adequate indicators of true recovery yields from actual CM resources. At the other end of the process, when evaluating cost, it is important to note that expenditure can come from other variables in addition to the basic production and operational costs (Fig. 6). Waste disposal may represent a significant expense especially, for instance, if radioactive metals (*e.g.*, uranium and thorium during REE extraction^60^) or other hazardous materials are also concentrated during the recovery process. Every SPE technology claiming to have real-world implications for CM extraction should justify that claim with recovery results from real feedstocks that are incorporated into a thorough techno-economic analysis. Indeed, the argument could be made that this economic performance should be the primary basis of comparison between SPE systems, rather than simply their fundamental metal extraction performance.

Finally, environmental considerations should not be neglected during SPE development. Extractive metallurgy has historically come with a steep environmental cost. Modern pyrometallurgical techniques, for example, can produce large volumes of high temperature off-gasses that would be harmful if released directly into the environment. On the other hand, hydrometallurgical techniques, like LLE, often require large volumes of potentially hazardous organic solvents, which again would be harmful if released into the environment. Even though strategies exist to mitigate the hazards, off-gases can be treated before being released and organic solvents can be contained and recycled, there are inherent environmental risks associated with these processes. Given that mining and metal extraction operations are frequently conducted in developing nations with limited oversight or regulation, it should not be taken for granted that the appropriate strategies for environmental impact mitigation are always employed. SPE could offer a competitive advantage in terms of minimizing environmental impact in two ways. First, it may enable CM recovery from non-traditional and recycled resources, limiting the necessity for CM extraction from primary resources; and second, the SPE process itself has the potential to have a relatively low environmental impact (Fig. 6). Researchers could do more to embrace these potential advantages by targeting non-traditional feedstocks and by actively investigating the environmental impact of their system. If it turns out that a highly engineered sorbent requires large volumes of hazardous chemicals for synthesis, for example, then the advantage over existing techniques may be diminished or lost entirely. The expected environmental impacts of developing SPE technologies should be assessed and reported, as they may represent a compelling benefit when compared to other methods. Emerging SPE technologies would benefit greatly from addressing scalability, economic viability, and environmental considerations even from the beginning of the development process.

## 5. Conclusions

Academia can provide essential contributions to advances in SPE for CM recovery, complementing the unfortunately often proprietary innovations coming from industry and governmental organizations; however, academic researchers should ensure that their work is firmly rooted in the practical necessities that motivate the research. Too often, assessments of scalability, economic feasibility, and environmental impact are ignored or indefinitely postponed pending further study. These oversights have resulted in a body of CM SPE literature that describes the varied sorptive characteristics of many promising materials, but which exhibits a frequent lack of connection to real-world applications. Of course, there are many groups that do focus on these vital facets of SPE research, often collaborating with industry and governmental partners and even advancing their systems towards large-scale use. A few promising examples of this progress include the West Virginia Water Research Institute (West Virginia University) pilot plant for CM recovery from acid mine drainage^10^ and the University of Kentucky pilot plant for REE extraction from coal and coal by-products,^14^ both of which are working in collaboration with government and industry partners. This application-driven practice should become standard in the field across the spectrum of technology maturity levels. Ultimately, academic researchers have made strides towards the development of practical, industrial-scale solid-phase extraction systems for CM recovery and purification. These SPE systems can offer numerous and important advantages in terms of cost, flexible performance, and environmental impact moving into the future, and increased focus on the real-world application of these technologies would facilitate further innovation.

## Data availability statement

Data sharing is not applicable to this article as no new data were created or analyzed in this study.

## Conflicts of interest

The authors have no conflicts to disclose (initial submission on 19.10.2021).

## Supplementary Material
